# CSF Rhinorrhea After Endonasal Intervention to the Skull Base (CRANIAL) — Part 2: Impact of COVID-19

**DOI:** 10.1016/j.wneu.2020.12.169

**Published:** 2021-05

**Authors:** CRANIAL Consortium, Soham Bandyopadhyay, Danyal Z. Khan, Hani J. Marcus, Benjamin E. Schroeder, Vikesh Patel, Alice O'Donnell, Shahzada Ahmed, Andrew F. Alalade, Ahmad M.S. Ali, Callum Allison, Sinan Al-Barazi, Rafid Al-Mahfoudh, Meriem Amarouche, Anuj Bahl, David Bennett, Raj Bhalla, Pragnesh Bhatt, Alexandros Boukas, Ivan Cabrilo, Annabel Chadwick, Yasir A. Chowdhury, David Choi, Simon A. Cudlip, Neil Donnelly, Neil L. Dorward, Graham Dow, Daniel M. Fountain, Joan Grieve, Anastasios Giamouriadis, Catherine Gilkes, Kanna Gnanalingham, Jane Halliday, Brendan Hanna, Caroline Hayhurst, Jonathan Hempenstall, Duncan Henderson, Kismet Hossain-Ibrahim, Theodore Hirst, Mark Hughes, Mohsen Javadpour, Alistair Jenkins, Mahmoud Kamel, Richard J. Mannion, Angelos G. Kolias, Mohammad Habibullah Khan, Mohammad Saud Khan, Peter Lacy, Shumail Mahmood, Eleni Maratos, Andrew Martin, Nijaguna Mathad, Patrick McAleavey, Nigel Mendoza, Christopher P. Millward, Showkat Mirza, Sam Muquit, Daniel Murray, Paresh P. Naik, Ramesh Nair, Claire Nicholson, Alex Paluzzi, Omar Pathmanaban, Dimitris Paraskevopoulos, Jonathan Pollock, Nick Phillips, Rory J. Piper, Bhaskar Ram, Iain Robertson, Elena Roman, Peter Ross, Thomas Santarius, Parag Sayal, Jonathan Shapey, Rishi Sharma, Simon Shaw, Alireza Shoakazemi, Syed Shumon, Saurabh Sinha, Georgios Solomou, Wai Cheong Soon, Simon Stapleton, Patrick Statham, Benjamin Stew, Nick Thomas, Georgios Tsermoulas, James R. Tysome, Adithya Varma, Philip Weir, Adam Williams, Mohamed Youssef, Damjan Veljanoski

**Affiliations:** 1 Medical Sciences Division, University of Oxford, Oxford, United Kingdom; 2 Wellcome/EPSRC Centre for Interventional and Surgical Sciences and National Hospital for Neurology and Neurosurgery, London, United Kingdom; 3 School of Medicine, Cardiff University, Cardiff, United Kingdom; 4 Division of Neurosurgery, Department of Clinical Neurosciences, Cambridge University Hospitals Trust, Cambridge, United Kingdom; 5 Birmingham Medical School, University of Birmingham, Birmingham, United Kingdom; 6 Department of Ear, Nose, and Throat, University Hospitals Birmingham NHS Foundation Trust, Birmingham, United Kingdom; 7 Department of Neurosurgery, Lancashire Teaching Hospitals NHS Foundation Trust, Lancashire, United Kingdom; 8 Department of Neurosurgery, The Walton Centre, Liverpool, United Kingdom; 9 Department of Neurosurgery, Royal Victoria Infirmary, Newcastle, United Kingdom; 10 Department of Neurosurgery, King's College Hospital, London, United Kingdom; 11 Department of Neurosurgery, Hurstwood Park Neurosciences Centre and Royal Sussex County Hospital, Haywards Heath, United Kingdom; 12 Department of Neurosurgery, John Radcliffe Hospital, Oxford University Hospitals NHS Foundation Trust, Oxford, United Kingdom; 13 Department of Neurosurgery, Hull University Teaching Hospitals, Hull, United Kingdom; 14 Department of Neurosurgery, Ninewells Hospital, Dundee, United Kingdom; 15 Department of Ear, Nose, and Throat, Salford Royal NHS Foundation Trust, Salford, United Kingdom; 16 Department of Neurosurgery, Aberdeen Royal Infirmary, Aberdeen, United Kingdom; 17 National Hospital for Neurology and Neurosurgery, London, United Kingdom; 18 Department of Neurosurgery, Manchester Centre for Clinical Neurosciences, Salford Royal Trust, Salford, United Kingdom; 19 Department of Neurosurgery, Queen Elizabeth Hospital Birmingham, Birmingham, United Kingdom; 20 Department of Ear, Nose, and Throat, Cambridge University Hospitals Trust, Cambridge, United Kingdom; 21 Department of Neurosurgery, Queen's Medical Centre Nottingham, Nottingham, United Kingdom; 22 Department of Ear, Nose, and Throat, Royal Victoria Hospital, Belfast, United Kingdom; 23 Department of Neurosurgery, University Hospital of Wales, Cardiff, United Kingdom; 24 Department of Neurosurgery, University Hospital Southampton, Southampton, United Kingdom; 25 Department of Neurosurgery, Royal Hallamshire Hospital & Sheffield Children's Hospital, Sheffield, United Kingdom; 26 Department of Neurosurgery, Royal Victoria Hospital, Belfast, United Kingdom; 27 Department of Neurosurgery, The Western General Hospital, Edinburgh, United Kingdom; 28 Department of Neurosurgery, National Neurosurgical Centre, Beaumont Hospital, Dublin, United Kingdom; 29 Department of Neurosurgery, Cork University Hospitals, Cork, United Kingdom; 30 Department of Ear, Nose, and Throat, Cork University Hospitals, Cork, United Kingdom; 31 Department of Ear, Nose, and Throat, National Neurosurgical Centre, Beaumont Hospital, Dublin, United Kingdom; 32 Department of Neurosurgery, St George's University Hospitals Trust, London, United Kingdom; 33 Department of Neurosurgery, Charing Cross Hospital, London, United Kingdom; 34 Department of Ear, Nose, and Throat, Sheffield Teaching Hospitals, Sheffield, United Kingdom; 35 Department of Neurosurgery, University Hospitals Plymouth, Plymouth, United Kingdom; 36 Department of Neurosurgery, Barts and The Royal London Hospital, London, United Kingdom; 37 Department of Neurosurgery, Barking, Havering and Redbridge University Hospitals, Romford, United Kingdom; 38 Department of Ear, Nose, and Throat, Aberdeen Royal Infirmary, Aberdeen, United Kingdom; 39 Department of Ear, Nose, and Throat, Ninewells Hospital, Dundee, United Kingdom; 40 Department of Neurosurgery, Royal Stoke University Hospital, Stoke-on-Trent, United Kingdom; ^41^School of Medicine, Keele University, Stoke-on-Trent, United Kingdom; 42 Department of Ear, Nose, and Throat, University Hospital of Wales, Cardiff, United Kingdom; 43 Department of Neurosurgery, Southmead Hospital Bristol, Bristol, United Kingdom; 44 Department of Neurosurgery, Leeds Teaching Hospitals NHS Trust, Leeds, United Kingdom

**Keywords:** Cerebrospinal fluid leak, Cerebrospinal fluid rhinorrhea, CSF, EEA, Endoscopic endonasal, Skull base surgery, CI, Confidence interval, COVID-19, Coronavirus disease 2019, CRANIAL, CSF rhinorrhea after endonasal intervention to the skull base, CSF, Cerebrospinal fluid, EEA, Expanded endoscopic endonasal approach, HCW, Healthcare worker, PPE, Personal protective equipment, SARS-CoV-2, Severe acute respiratory syndrome coronavirus 2, TSA, Transsphenoidal approach

## Abstract

**Background:**

During the coronavirus disease 2019 (COVID-19) pandemic, concerns have been raised regarding the increased risk of perioperative mortality for patients with COVID-19, and the transmission risk to healthcare workers, especially during endonasal neurosurgical operations. The Pituitary Society has produced recommendations to guide management during this era. We sought to assess contemporary neurosurgical practice and the effects of COVID-19.

**Methods:**

A multicenter prospective observational cohort study was conducted at 12 tertiary neurosurgical units (United Kingdom and Ireland). Data were collected from March 23 to July 31, 2020, inclusive. The data points collected included patient demographics, preoperative COVID-19 test results, operative modifications, and 30-day COVID-19 infection rates.

**Results:**

A total of 124 patients were included. Of the 124 patients, 116 (94%) had undergone COVID-19 testing preoperatively (transsphenoidal approach, 97 of 105 [92%]; expanded endoscopic endonasal approach, 19 of 19 [100%]). One patient (1 of 116 [0.9%]) had tested positive for COVID-19 preoperatively, requiring a delay in surgery until the infection had been confirmed as resolved. Other than transient diabetes insipidus, no other complications were reported for this patient. All operating room staff had worn at least level 2 personal protective equipment. Adaptations to surgical techniques included minimizing drilling, draping modifications, and the use of a nasal iodine wash. At 30 days postoperatively, no evidence of COVID-19 infection (symptoms or positive formal testing results) were found in our cohort and no mortality had occurred.

**Conclusions:**

Preoperative screening protocols and operative modifications have facilitated endonasal neurosurgery during the COVID-19 pandemic, with the Pituitary Society guidelines followed for most of these operations. We found no evidence of COVID-19 infection in our cohort and no mortality, supporting the use of risk mitigation strategies to continue endonasal neurosurgery in subsequent pandemic waves.

## Introduction

The coronavirus disease 2019 (COVID-19) pandemic is an ongoing global pandemic caused by a novel coronavirus (severe acute respiratory syndrome coronavirus 2 [SARS-CoV-2]).^1^^,^^2^ Measures were enacted to mitigate the spread of the virus and, thereby, prevent national healthcare systems from being overwhelmed. These measures included reenlisting retired healthcare workers^3^ and redeploying surgeons to provide out-of-specialty care.^4^^,^^5^ As a secondary consequence of this reallocation of resources in healthcare services globally, it proved increasingly challenging to continue providing existing services for other diseases and conditions, including a noticeable reduction in surgical activity.^6^, ^7^, ^8^ This reduction was likely compounded by the initial concern that patients undergoing surgery would be an especially vulnerable group owing to their risk of exposure^9^ and, as such, a more cautious approach was taken regarding surgery.

One such specialty that has seen their normal services disrupted is neurosurgery,^10^ with some countries reporting the cancellation of more than one half of all their indicated neurosurgical operations.^11^ This has been especially so for pituitary surgery.^12^^,^^13^ One factor that played a role in the reduction of pituitary surgery was the need to protect healthcare workers (HCWs).^10^^,^^12^ The transmission of the SARS-CoV-2 virus occurs primarily via large respiratory droplets containing the virus. Therefore, HCWs working in certain specialties and subspecialties were considered to have a high risk owing to their frequent exposure to oronasal secretions.^14^ These HCWs include those involved in pituitary surgery, given the number of procedures for which access is via the nasal cavity and sphenoid sinus.^15^ To manage the risk to HCWs involved in such procedures, the Professional Education Committee of the Pituitary Society produced a set of comprehensive guidelines for international guidance.^12^ These included advising all patients to undergo COVID-19 screening preoperatively, the use of non-drill techniques, considerations for alternative approaches (e.g., transcranial), and for operating room staff involved in endoscopic or microscopic endonasal approach surgeries to wear at least level 2 personal protective equipment (PPE).^16^^,^^17^

Although the caseloads in neurosurgery have decreased, some operations have been performed during the course of the pandemic.^18^^,^^19^ These operations were most likely performed for patients requiring urgent or emergency surgery,^20^ including patients presenting with pituitary apoplexy, visual loss, malignant pathology, or significant endocrine disorders. However, reported data are lacking regarding the number of neurosurgical operations performed, their indications, and the postoperative complications for the patients who had undergone neurosurgical operations during the COVID-19 pandemic. These data are essential, especially to quantify the effects on those who have undergone an endonasal operation in the context of a respiratory virus. The timeline of a prospective multicenter pilot study in the United Kingdom and Republic of Ireland—CRANIAL (CSF [cerebrospinal fluid] rhinorrhea after endonasal intervention to the skull base)^21^—coincided with the start of the COVID-19 pandemic. This provided a unique opportunity to assess the effects of the COVID-19 pandemic on endonasal surgery practices in the United Kingdom in real time. Therefore, in the present report, our primary aim was to determine whether the advice regarding COVID-19 testing and the use of PPE had been followed, and to investigate the intraoperative adaptations used to minimize the risk of COVID-19, the 30-day postoperative COVID-19 infection rate, and the mortality rate.

## Methods

### Study Design

A multicenter prospective observational cohort study design was implemented^21^ at 12 tertiary academic neurosurgical units where the CRANIAL network had previously been established: Aberdeen Royal Infirmary (Aberdeen, United Kingdom), Addenbrooke's Hospital (Cambridge, United Kingdom), Beaumont Hospital (Dublin, Ireland), Greater Manchester Neurosciences Centre (Salford, United Kingdom), John Radcliffe Hospital (Oxford, United Kingdom), National Hospital for Neurology and Neurosurgery (London, United Kingdom), Queen Elizabeth Hospital Birmingham (Birmingham, United Kingdom), Royal Hallamshire Hospital (Sheffield, United Kingdom), Royal Victoria Hospital (Belfast, United Kingdom), Royal Victoria Infirmary (Newcastle-upon-Tyne, United Kingdom), Sheffield Children's Hospital (Sheffield, United Kingdom), and the Walton Centre (Liverpool, United Kingdom). The project was registered as a service evaluation at each center, with approval received from the audit department of each institution (and Caldicott guardians when required). The local team consisted of consultant lead(s) with overall project responsibility, trainee lead(s) in charge of data collection, and, on occasion, student lead(s) for additional support. The STROBE (strengthening the reporting of observational studies in epidemiology) statement was used in the preparation of this report.^22^

The eligible cases included patients of all ages who had undergone the transsphenoidal approach (TSA) for sellar tumors or the expanded endonasal approach (EEA) for skull base tumors.^21^ The exclusion criteria were transcranial surgery and a history of preoperative CSF rhinorrhea. Case selection was limited to patients who had presented from March 23 to July 31, 2020, inclusive. Before March 23, 2020, pauses had occurred in data collection owing to data pro forma amendments and attaining additional approvals (e.g., information governance approvals where requested). This also allowed for our data to coincide with when the first lockdown had begun in the United Kingdom.^23^

### Data Collection

The data points collected included the patient demographics, preoperative COVID-19 status, operative modifications, and postoperative COVID-19 data. The primary outcomes of interest were as follows: 1) the COVID-19 preoperative screening method used; 2) the precautions taken to reduce the risk of airborne pathogen transmission; and 3) the 30-day COVID-19 infection rate for patients postoperatively. The secondary outcomes of interest were the length of hospital stay and the mortality rate.

The local teams submitted data to a secure web-based central database hosted by Castor Electronic Data Capture (Amsterdam, The Netherlands; available at: https://www.castoredc.com/). All initial data were collected within 30 days of surgery, followed by a 30-day follow-up window. The data points collected by medical students or junior trainees were confirmed with the operating surgeons or senior members of the team before final submission to the Castor Electronic Data Capture system.^21^

### Data Validation

Data validation was performed in all centers to audit quantitative data accuracy.^21^ This involved an independent data validator (who had not collected the local data) who reviewed the datasets for several enrolled cases, selected randomly. Each data validator was from the hospital in which the data had been collected. The targets for validation were a secure and accurate record of the Castor identification records with corresponding medical record numbers, no case or data duplication, and data accuracy of >95%.

### Statistical Analysis

The pooled quantitative data were analyzed using Microsoft Excel, version 16.41 (Microsoft, Redmond, Washington, USA), to present descriptive statistics. If a data point was missing from a case, the denominator was adjusted to account for the missing data. The data were used to create tables summarizing the demographic characteristics, tumor characteristics, operative characteristics, and methods used to reduce COVID-19 transmission. For 3 centers, data were available before the lockdown had begun (November 1, 2019 to March 22, 2020). The Mann-Whitney U test was used for comparative analysis (pre-lockdown vs. during lockdown) of patient age and hospital length of stay for the patients from these 3 centers. The Fisher exact test was for comparative analysis (pre-lockdown vs. during lockdown) of the remaining data points for patients from these 3 centers. Statistical analysis was performed using Prism, version 5 (GraphPad, San Diego, California, USA), with statistical significance set at P ≤ 0.05.

## Results

### General Data

Data were collected for 124 patients across the 12 tertiary neurosurgical centers. The neurosurgical centers had contributed data for 2 to 21 patients. No duplicates in the cases or data were found in the records audited for data validation. All centers had fulfilled the >95% accuracy target per case.

### Patient Characteristics

The median patient age in the present study was 50.5 years (range, 7–82 years). Of the 124 patients, 65 were male and 59 were female. At presentation, the body mass index had been recorded for 118 patients (95%). Of these 118 patients, 32 (27%) had had a body mass index >30 kg/m^2^ (29 of 96 in the TSA group [30%] and 3 of 22 in the EEA group [14%]). The patient's vision at presentation had been recorded for 121 of the 124 patients (98%). Visual loss (acuity and/or field deficits) was present in 83 of these 121 patients (69%) preoperatively (TSA, 69 of 99 [70%]; EEA, 14 of 22 [64%]). Of the 124 patients, 30 (24%) had presented with anterior pituitary deficiency requiring hydrocortisone therapy preoperatively (TSA, 25 of 102 [25%]; EEA, 5 of 22 [23%]). Five patients (4%) had had posterior pituitary deficiency requiring desmopressin preoperatively (TSA, 4 of 102 [4%]; EEA, 1 of 22 [5%]). These data are summarized in Table 1. Comparing the preoperative factors from before and during the lockdown at the 3 pilot centers, a larger proportion of patients who had undergone surgery during the lockdown had had visual compromise preoperatively compared with those treated before the lockdown (*P* ≤ 0.01; Table 2).

**Table 1:** Summary of Patient Demographics

Variable	Approach (n)		Total (n)
	TSA	EEA	
Total patients	102	22	124
Sex			
Male	54	11	65
Female	48	11	59
BMI (kg/m^2^)			
>30	29	3	32
<30	67	19	86
Visual loss			
Yes	69	14	83
No	30	8	38
Anterior pituitary deficiency requiring hydrocortisone preoperatively	25	5	30
Posterior pituitary deficiency requiring desmopressin preoperatively	4	1	5

TSA, transsphenoidal approach; EEA, expanded endonasal approach; BMI, body mass index.

**Table 2:** Comparison of Baseline Data Points and Postoperative Outcomes at 3 Centers Before and During COVID-19

Variable	Before Pandemic (n; % or Range)	During Pandemic (n; % or Range)	*P* Value
Total patients	60	45	
Preoperative			
Median age (range)	52.7 (18–84)	45 (8–82)	0.08∗
Visual loss	21 (35)	32 (71)	<0.01†‡
Anterior pituitary deficiency requiring hydrocortisone	12 (20)	12 (27)	0.49†
Posterior pituitary deficiency requiring desmopressin	1 (2)	2 (4)	0.58†
Tumor size >1 cm in diameter	49 (82)	41 (91)	0.26†
Operative time (minutes)			
Median (range)	83 (35–200)	80 (35–302)	0.28∗
Median TSA (range)	80 (35–195)	76 (35–230)	0.78∗
Median EEA (range)	151 (83–200)	259 (137–302)	0.21∗
Postoperative			
Median length of stay (days; range)	4 (1–20)	5 (1–20)	0.18∗
General complications	10 (17)	15 (33)	0.06†
CSF rhinorrhea (biochemically confirmed or requiring surgery)	3 (5)	4 (9)	0.46†
CSF rhinorrhea requiring surgery§	2 (3)	3 (7)	0.65†

COVID-19, coronavirus disease 2019; TSA, transsphenoidal approach; EEA, expanded endonasal approach; CSF, cerebrospinal fluid.

∗ Mann-Whitney *U* test.

† Fisher's exact test.

‡ Visual loss was a significantly more common complaint for patients presenting during the pandemic compared with before the pandemic.

§ CSF diversion or direct repair.

Most of the tumors were pituitary adenomas (*n* = 88 of 124; 71%), and most of these were macroadenomas (*n* = 82 of 88; 93%). Of the 124 tumors, 63 were nonfunctioning pituitary adenomas (51%). Of these 63 tumors, 62 were macroadenomas (98%). Of the 25 functioning pituitary adenomas (20% of the 124 tumors), 20 were macroadenomas (20 of 25; 80%). Of the patients with a functioning pituitary adenoma, 20 had had either Cushing disease (*n* = 10; 40%) or acromegaly/gigantism (*n* = 10; 40%). The remaining 36 pathological entities (29%) were ≥1 cm in size. The characteristics of the remaining tumors are presented in Table 3.

**Table 3:** Tumor Type Stratified by Surgical Approach

Tumor Type	TSA (n)	EEA (n)	Total (n)
Nonfunctioning pituitary adenoma	61	2	63
Functioning pituitary adenoma	24	1	25
Craniopharyngioma	4	9	13
Apoplexy	2	1	3
Rathke's cleft cyst	3	0	3
Chordoma	0	2	2
Cystic lesion (unspecified)	0	2	2
Meningioma	0	2	2
Arachnoid cyst	1	0	1
Germinoma	1	0	1
Hypophysitis	1	0	1
Melanoma metastasis	1	0	1
Meningoencephalocele	0	1	1
Mucinous gland	1	0	1
Prostate metastasis	1	0	1
Sinonasal carcinoma	0	1	1
Sinonasal endocrine tumor	1	0	1
Squamous cell carcinoma	0	1	1
Undefined neuroendocrine tumor	1	0	1

TSA, transsphenoidal approach; EEA, expanded endonasal approach.

### COVID-19 Screening Preoperatively

Preoperatively, 2 of the 124 patients (2%) had presented with symptoms associated with COVID-19: 1 with a new cough and 1 with shortness of breath. Neither of these patients had tested positive for COVID-19 when screened and both had eventually undergone endoscopic surgery with the TSA—1 patient after 2 weeks self-isolation and negative swab results and 1 after computed tomography of the thorax and swab testing were negative (without isolation owing to clinical urgency). Of the 124 patients, 116 (94%) had undergone COVID-19 testing preoperatively (TSA, 95 of 102 [93%]; EEA, 21 of 22 [95%]). Eight patients had not undergone COVID-19 testing preoperatively, usually because of clinical urgency and the lack of rapid testing facilities at the time (TSA, 7 of 102 [7%]; EEA, 1 of 22 [5%]). Of the patients who had undergone screening, a swab test was used for all 116 (100%), with 1 patient also screened using computed tomography of the thorax (0.9%). Of these 116 patients, 1 (0.9%) had tested positive for COVID-19 via the swab test preoperatively. The patient who had tested positive (a 52-year-old man) underwent self- isolation for 2 weeks and re-screening preoperatively. He subsequently had an endoscopic TSA for pituitary macroadenoma resection after a re-screening swab test result was negative. The swab types used were examined at the initial pilot centers (*n* = 4) and were either RNA polymerase chain reaction tests (*n* = 2 of 4) or RNA transcription-mediated amplification tests (*n* = 2 of 4).

### Operation Characteristics

**Figure 1:**
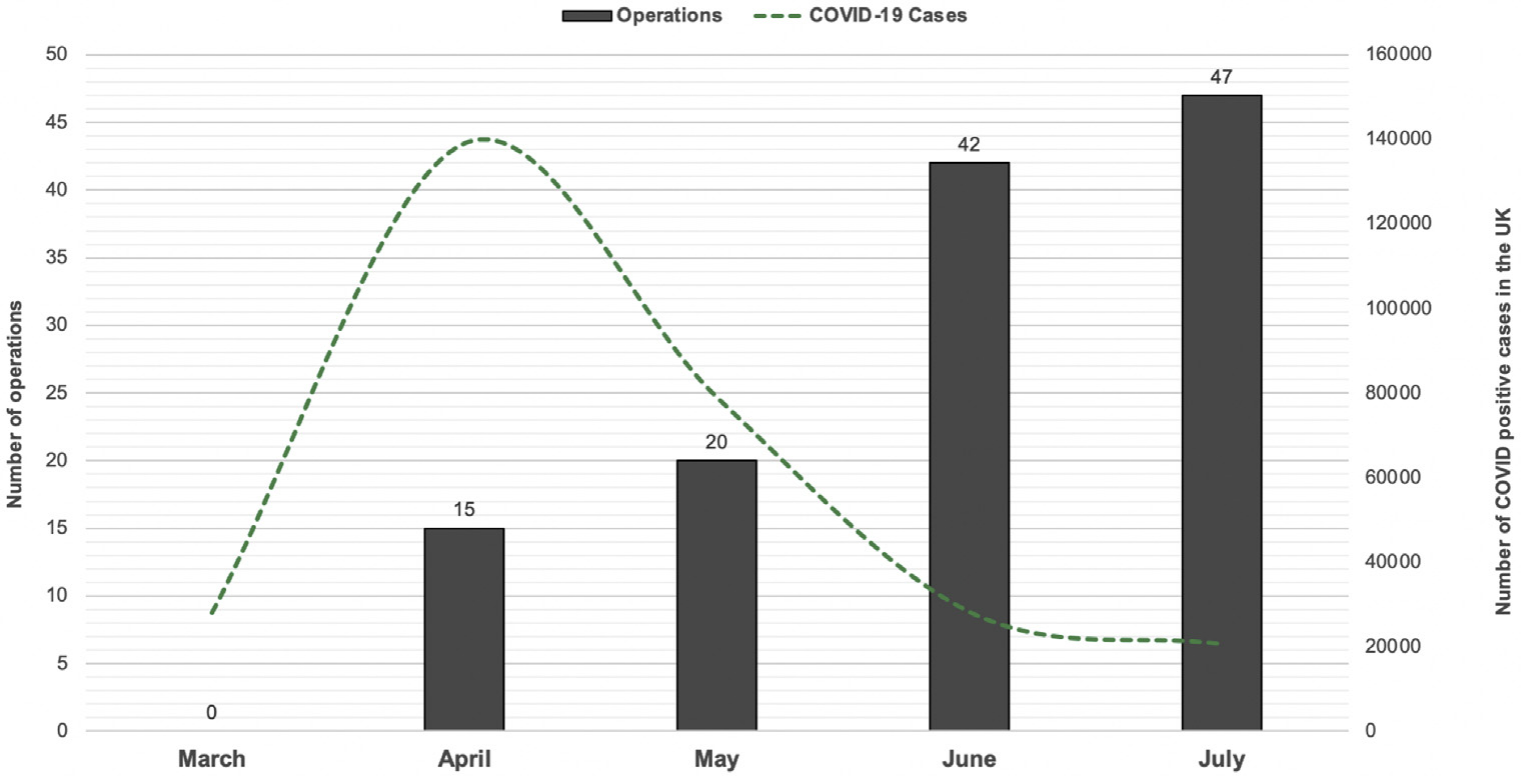
Bar graph showing the number of operations per month with an overlay of the number of coronavirus disease 2019 (COVID-19) cases in the United Kingdom during the study period (data extracted from the Public Health England database). No operations were reported from March 23 to March 31, 2020.

Most of the operations (*n* = 47 of 124; 38%) were performed in July (Figure 1). The median caseload during the pandemic was 44.8% (interquartile range, 39.4%–49.3%) of the usual operative volume when compared with the caseload during the same period in 2019 at selected core pilot centers (*n* = 3).

Most of the cases had used the TSA (*n* = 102 of 124; 82%). Of the 102 TSA cases, 91 were performed endoscopically (89%) and 11 microscopically (11%). Also, of the 102 TSA cases, 92 were the primary surgery (90%). The most common pathological entities treated using the TSA included nonfunctioning pituitary adenomas (*n* = 61), functioning pituitary adenomas (*n* = 24), and craniopharyngiomas (*n* = 4; Table 3). The EEA was used for 22 of the 124 cases (18%). The most common pathological entities treated using the EEA included craniopharyngiomas (*n* = 9), meningiomas (*n* = 2), chordomas (*n* = 2), and nonfunctioning pituitary adenomas (*n* = 2; Table 3). Of the 22 EEA cases, 20 were the primary surgery (91%).

The operating room staff involved in the 124 operations had worn a range of PPE. The PPE used included surgical face masks (TSA, 2 of 102 [2%]; EEA, 0 of 22 [0%]), FFP3 masks (TSA, 82 of 102 [80%]; EEA, 17 of 22 [77%]), powered hood respirators (TSA, 45 of 102 [44%]; EEA, 10 of 22 [45%]), eyeglasses (TSA, 59 of 102 [58%]; EEA, 15 of 22 [68%]), face shields (TSA, 47 of 102 [46%]; EEA, 10 of 22 [45%]), standard surgical gowns (TSA, 59 of 102 [58%]; EEA, 12 of 22 [55%]), double surgical gowns (TSA, 2 of 102 [2%]; EEA, 0 of 22 [0%]), and reinforced surgical gowns (TSA, 26 of 102 [25%]; EEA, 5 of 22 [23%]). Additional measures to reduce the risk of airborne transmission are listed in Table 4.

**Table 4:** Additional Measures Enacted by Neurosurgical Centers to Reduce Risk of Airborne Transmission of COVID-19

Modification
Preoperative
Patients isolated for 2 weeks preoperatively
Reduction in number of staff in operating room
Most operating room staff restricted from entering operating room until 10 minutes after intubation
Intraoperative
Patient covered with clear plastic cover over regular drape
Instruments sealed with tape and plastic drapes
Use of 9 mL of 0.5% PVP-I solution for skin and mucous membranes as mouth wash
Instillation of 0.3 mL of 0.5% PVP-I solution for skin and mucous membranes in each nostril
Change from fluoroscopy to Stealth∗ to decrease movement of equipment through multiple operating rooms
Minimization of bone drilling
Postoperative
Nasal packing avoided whenever possible
Most operating room staff not present during extubation

COVID-19, coronavirus disease 2019; PVP-I, povidone-iodine.

∗ StealthStation, Medtronic, Dublin, Ireland.

### Postoperative Complications and Screening

The median length of patient stay was 4 days (range, 1–32 days) for the entire group and was 3.5 days (range, 1–32 days) for the TSA group and 7 days (range, 1–20 days) for the EEA group. Overall, 28 of the 124 patients (23%) had experienced postoperative complications (TSA, 19 of 102 [19%]; EEA, 9 of 22 [41%]). The most common complications were diabetes insipidus (TSA, 7 of 102 [7%]; EEA, 4 of 22 [18%]), postoperative CSF rhinorrhea (TSA, 4 of 102 [4%]; EEA, 2 of 22 [9%]), and syndrome of inappropriate antidiuretic hormone secretion (TSA, 4 of 102 [4%]; EEA, 1 of 22 [5%]). Other complications that had occurred in the TSA group included residual disease, meningitis, sellar abscess, unspecified hyponatremia, and mono-ocular blindness in 1 patient each (1%). The other complications that had occurred in the EEA group included residual disease in 2 (9%) and unspecified hyponatremia in 1 (5%). None of the 124 patients had died within 30 days postoperatively. The 1 patient with a recent history of COVID-19 infection had had transient diabetes insipidus postoperatively but no concerns for postoperative COVID-19 infection or respiratory compromise. No significant differences were found in postoperative outcomes when comparing the postoperative outcomes before and during the lockdown at the 3 pilot centers (Table 2).

Of the 124 patients, COVID-19 data at 30 days postoperatively were available for 114 patients (92%). Postoperatively, 1 patient (0.9%) had presented with symptoms associated with COVID-19: a new cough. This patient had not tested positive for COVID-19 when screened. Of the 124 patients, 19 (15%) had undergone COVID-19 screening within 30 days postoperatively (TSA, 11 of 102 [11%]; EEA, 8 of 22 [36%]). All 19 patients had undergone screening using the swab test, and none had tested positive for COVID-19. Data on the staff involved in surgery were available for 48 cases only. None of the staff had tested positive for COVID-19 within 30 days postoperatively.

## Discussion

### Principal Findings

To the best of our knowledge, the present study is the first multicenter study reporting data of contemporaneous endonasal skull base operative practice during the COVID-19 pandemic. As expected, the operative caseload was lowest at the peak of the pandemic (March) and increased over time as operative protocols were established and infection rates reduced. Most of these endonasal neurosurgical cases were for pituitary adenoma (n = 88 of 124 [71%]), and most patients were symptomatic preoperatively: visual loss in 83 of 121 patients (69%), anterior pituitary deficiency requiring hydrocortisone in 30 of 124 patients (24%), and/or posterior pituitary deficiency requiring desmopressin in 5 patients (4%). The most common approach used was the TSA (n = 102 of 124; 82%). The operating room staff involved in these operations adhered to international guidance by wearing at least level 2 PPE.^12^ Considerable heterogeneity was present in the PPE worn; however, the PPE items worn in most cases were FFP3 masks (*n* = 99 of 124; 80%) and eyeglasses (*n* = 74 of 124; 60%). Adaptations to surgical techniques included minimizing drilling, draping modifications, and using nasal iodine wash.

At 30 days postoperatively, no evidence was found of COVID-19 infection (no symptoms and no positive test results) in our cohort and no mortality had occured. One of the 116 patients had tested positive for COVID-19 preoperatively and was isolated for 2 weeks, with negative swab screening results before the patient underwent surgery. This patient had developed transient diabetes insipidus postoperatively; however no other complications had been reported at 30 days postoperatively.

### Findings in the Context of the Reported Data

Few studies have provided data on patients who have undergone pituitary surgery during the COVID-19 pandemic. One case report from Wuhan, China, described a patient developing COVID-19 within the first week after endoscopic endonasal pituitary surgery. However, the use of a preoperative swab screening test was not reported; thus, it was unclear whether the patient had had COVID-19 preoperatively or had developed a postoperative infection.^19^ Additionally, a case series from Cambridge, United Kingdom, reported that none of 9 consecutive patients who had undergone pituitary surgery or skull base surgery from March 30 to April 28, 2020, had contracted COVID-19 after the adoption of a risk-mitigation protocol.^12^, ^18^ Similar risk-mitigation strategies were subsequently advocated by the Professional Education Committee of the Pituitary Society. The results from our international, multicenter study support the findings of the latter study, because we did not find a greater risk to patients of acquiring COVID-19 if they underwent endonasal surgery during the pandemic. Our results also suggest that a standardized, risk-mitigation strategy that accounts for earlier guidance might allow for normalization of activity. Our results join an increasing body of data showing that surgery is safe for patients with negative SARS-CoV-2 test results preoperatively in a COVID-19–free surgical pathway.^24^^,^^25^ The preoperative swab tests used in our series were RNA polymerase chain reaction and transcription-mediated amplification via nasopharyngeal swabs. The use of a single swab is estimated to have a sensitivity of 70% and specificity of 95%—with the true predictive value determined by factors such as symptoms and disease prevalence.^12^^,^^26^^,^^27^ However, the resumption of full elective workloads will depend on wider national and international factors that protect patients from becoming infected with SARS-CoV-2 and, thereby, avoid delays in surgery. The non–COVID-19 morbidity of patients with pituitary pathology is an increasing concern, and our results could help alleviate concerns about performing surgery during the pandemic.

### Study Limitations

The present study had several limitations, which require a tempered assessment of the findings. Firstly, because of the reduced caseloads aimed at mitigating the effects of COVID-19, we had a moderate sample size, especially with respect to EEA. Owing to the recency of cases and the urgency to inform policymakers about the risks to surgical patients, the follow-up period was limited to the 30-day postoperative period. In addition, because the present study was a prospective cohort study, the data points were purely observational and across the context of multiple centers. Also, owing to the observational nature, not all patients had been screened postoperatively for COVID-19, and we were reliant on these patients self-reporting any COVID-19 symptoms if and when they had developed. Similarly, robust and consistent data regarding the symptoms or infection rates of the surgical team were lacking. The most significant limitation was the paucity of baseline data to compare against the postoperative findings. The prepandemic dataset used in the present study for limited comparative analysis was not consecutive and was small in size. The main CRANIAL study is ongoing and should provide the baseline data with which to correlate our findings once the study has been completed.^21^

## Conclusions

As operative protocols were established and infection rates reduced, the number of endonasal operations increased. Guidelines reported by the Professional Education Committee of the Pituitary Society were followed. No postoperative COVID-19 infections occurred; therefore, the patients who had undergone surgery had not experienced any COVID-19–related morbidity or mortality. This suggests that a risk mitigation approach can enable timely pituitary surgery to continue safely during the COVID-19 pandemic.

## CRediT authorship contribution statement

**Soham Bandyopadhyay:** Resources, Data curation, Formal analysis, Writing - original draft, Writing - review & editing. **Danyal Z. Khan:** Conceptualization, Resources, Data curation, Formal analysis, Writing - original draft, Writing - review & editing. **Hani J. Marcus:** Conceptualization, Formal analysis, Writing - original draft, Writing - review & editing, Supervision. **Benjamin E. Schroeder:** Formal analysis, Writing - original draft, Writing - review & editing. **Vikesh Patel:** Resources, Data curation, Formal analysis, Writing - original draft, Writing - review & editing. **Alice O'Donnell:** Writing - original draft, Writing - review & editing. **Shahzada Ahmed:** Resources, Writing - review & editing, Writing - review & editing. **Andrew F. Alalade:** Resources, Writing - review & editing. **Ahmad M.S. Ali:** Resources, Data curation, Writing - review & editing. **Callum Allison:** Resources, Data curation, Writing - review & editing. **Rafid Al-Mahfoudh:** Resources, Writing - review & editing. **Meriem Amarouche:** Resources, Writing - review & editing. **Anuj Bahl:** Resources, Writing - review & editing. **David Bennett:** Resources, Writing - review & editing. **Raj Bhalla:** Resources, Writing - review & editing. **Pragnesh Bhatt:** Resources, Writing - review & editing. **Alexandros Boukas:** Resources, Data curation, Writing - review & editing. **Ivan Cabrilo:** Resources, Data curation, Writing - review & editing. **Annabel Chadwick:** Resources, Data curation, Writing - review & editing. **Yasir A. Chowdhury:** Conceptualization, Resources, Data curation, Writing - review & editing. **David Choi:** Resources, Writing - review & editing. **Simon A. Cudlip:** Resources, Writing - review & editing. **Neil Donnelly:** Resources, Writing - review & editing. **Neil L. Dorward:** Resources, Writing - review & editing. **Graham Dow:** Resources, Writing - review & editing. **Daniel M. Fountain:** Conceptualization, Resources, Data curation, Writing - review & editing. **Joan Grieve:** Resources, Writing - review & editing. **Anastasios Giamouriadis:** Resources, Writing - review & editing. **Catherine Gilkes:** Resources, Writing - review & editing. **Kanna Gnanalingham:** Resources, Writing - review & editing. **Jane Halliday:** Resources, Writing - review & editing. **Brendan Hanna:** Resources, Writing - review & editing. **Caroline Hayhurst:** Resources, Writing - review & editing. **Jonathan Hempenstall:** Resources, Writing - review & editing. **Duncan Henderson:** Resources, Data curation, Writing - review & editing. **Kismet Hossain-Ibrahim:** Resources, Writing - review & editing. **Theodore Hirst:** Resources, Data curation, Writing - review & editing. **Mark Hughes:** Resources, Writing - review & editing. **Mohsen Javadpour:** Resources, Writing - review & editing. **Alistair Jenkins:** Resources, Writing - review & editing. **Mahmoud Kamel:** Resources, Writing - review & editing. **Richard J. Mannion:** Resources, Writing - review & editing. **Angelos G. Kolias:** Conceptualization, Resources, Data curation, Writing - review & editing. **Mohammad Habibullah Khan:** Resources, Writing - review & editing. **Mohammad Saud Khan:** Resources, Data curation, Writing - review & editing. **Peter Lacy:** Resources, Writing - review & editing. **Shumail Mahmood:** Resources, Data curation, Writing - review & editing. **Eleni Maratos:** Resources, Writing - review & editing. **Andrew Martin:** Resources, Writing - review & editing. **Nijaguna Mathad:** Resources, Writing - review & editing. **Patrick McAleavey:** Resources, Data curation, Writing - review & editing. **Nigel Mendoza:** Resources, Writing - review & editing. **Christopher P. Millward:** Resources, Data curation, Writing - review & editing. **Showkat Mirza:** Resources, Writing - review & editing. **Sam Muquit:** Resources, Writing - review & editing. **Daniel Murray:** Resources, Data curation, Writing - review & editing. **Paresh P. Naik:** Resources, Data curation, Writing - review & editing. **Ramesh Nair:** Resources, Writing - review & editing. **Claire Nicholson:** Resources, Writing - review & editing. **Alex Paluzzi:** Resources, Writing - review & editing. **Omar Pathmanaban:** Resources, Writing - review & editing. **Dimitris Paraskevopoulos:** Resources, Writing - review & editing. **Jonathan Pollock:** Resources, Writing - review & editing. **Nick Phillips:** Resources, Writing - review & editing. **Rory J. Piper:** Resources, Writing - review & editing. **Bhaskar Ram:** Resources, Writing - review & editing. **Iain Robertson:** Resources, Writing - review & editing. **Elena Roman:** Resources, Data curation, Writing - review & editing. **Peter Ross:** Resources, Writing - review & editing. **Thomas Santarius:** Resources, Writing - review & editing. **Parag Sayal:** Resources, Writing - review & editing. **Jonathan Shapey:** Resources, Writing - review & editing. **Rishi Sharma:** Resources, Writing - review & editing. **Simon Shaw:** Resources, Writing - review & editing. **Alireza Shoakazemi:** Resources, Writing - review & editing. **Syed Shumon:** Resources, Data curation, Writing - review & editing. **Saurabh Sinha:** Resources, Writing - review & editing. **Georgios Solomou:** Resources, Writing - review & editing. **Wai Cheong Soon:** Resources, Data curation, Writing - review & editing. **Simon Stapleton:** Resources, Writing - review & editing. **Patrick Statham:** Resources, Writing - review & editing. **Benjamin Stew:** Resources, Writing - review & editing. **Nick Thomas:** Resources, Writing - review & editing. **Georgios Tsermoulas:** Resources, Writing - review & editing. **James R. Tysome:** Resources, Writing - review & editing. **Adithya Varma:** Resources, Data curation, Writing - review & editing. **Damjan Veljanoski:** Resources, Data curation, Writing - review & editing. **Philip Weir:** Resources, Writing - review & editing. **Adam Williams:** Resources, Writing - review & editing. **Mohamed Youssef:** Resources, Writing - review & editing.

## References

[1] World Health Organization WHO Director-General’s opening remarks at the media briefing on COVID-19—11 March 2020. https://www.who.int/dg/speeches/detail/who-director-general-s-opening-remarks-at-the-media-briefing-on-covid-19---11-march-2020.

[2] Zhu N., Zhang D., Wang W. (2020). A novel coronavirus from patients with pneumonia in China, 2019. N Engl J Med.

[3] Retired Doctors Return to Work for COVID-19. https://www.nextavenue.org/retired-doctors-return-to-work-for-covid-19/.

[4] BMA Redeploying staff, working in hubs and furlough—COVID-19: toolkit for GPs and GP practices. https://www.bma.org.uk/advice-and-support/covid-19/gp-practices/covid-19-toolkit-for-gps-and-gp-practices/redeploying-staff-working-in-hubs-and-furlough.

[5] Deployment of Surgeons for Out-of-Specialty Patient Care. https://www.facs.org/covid-19/clinical-guidance/workforce-deployment.

[6] Shehata I.M., Elhassan A., Jung J.W., Urits I., Viswanath O., Kaye A.D. (2020). Elective cardiac surgery during the COVID-19 pandemic: proceed or postpone?. Best Pract Res Clin Anaesthesiol.

[7] Gaudino M., Chikwe J., Hameed I., Robinson N.B., Fremes S.E., Ruel M. (2020). Response of cardiac surgery units to COVID-19: an internationally-based quantitative survey. Circulation.

[8] Cano-Valderrama O., Morales X., Ferrigni C.J. (2020). Reduction in emergency surgery activity during COVID-19 pandemic in three Spanish hospitals. Br J Surg.

[9] Besnier E., Tuech J.-J., Schwarz L. (2020). We asked the experts: COVID-19 outbreak: is there still a place for scheduled surgery? “Reflection from pathophysiological data. World J Surg.

[10] Hill C.S., Muirhead W.R., Vakharia V.N., Marcus H.J., Choi D. (2020). An exit strategy for resuming nonemergency neurosurgery after severe acute respiratory syndrome coronavirus 2: a United Kingdom perspective. World Neurosurg.

[11] El-Ghandour N.M.F., Elsebaie E.H., Salem A.A. (2020). Letter: the impact of the coronavirus (COVID-19) pandemic on neurosurgeons worldwide. Neurosurgery.

[12] Fleseriu M., Buchfelder M., Cetas J.S. (2020). Pituitary society guidance: pituitary disease management and patient care recommendations during the COVID-19 pandemic—an international perspective. Pituitary.

[13] Amin-Hanjani S., Bambakidis N.C., Barker F.G. (2020). COVID-19 and neurosurgical practice: an interim report. J Neurosurg.

[14] Edsel B., Xu A., Salimi A., Torun N. (2020). Physician deaths from coronavirus disease (COVID-19). Occup Med (Lond).

[15] Patel Z.M., Fernandez-Miranda J., Hwang P.H. (2020). Letter: precautions for endoscopic transnasal skull base surgery during the COVID-19 pandemic. Neurosurgery.

[16] Health Protection Scotland Appendix 16—Best Practice—Aide Memoire for Levels of Personal Protective Equipment (PPE) for Healthcare Workers When Providing Patient Care. http://www.nipcm.hps.scot.nhs.uk/media/1437/2019-02-11-aide-memoire-for-levels-of-personal-protective-equipment-ppe-for-healthcare-workers-for-patient-care.pdf.

[17] Khan K. Pitch side emergency care and personal protective equipment: a framework for elite sport during the COVID-19 pandemic: part 1 of 3. BJSM blog—social media’s leading SEM voice. https://blogs.bmj.com/bjsm/2020/07/08/pitch-side-emergency-care-personal-protective-equipment/.

[18] Kolias A., Tysome J., Donnelly N. (2020). A safe approach to surgery for pituitary and skull base lesions during the COVID-19 pandemic. Acta Neurochir (Wien).

[19] Zhu W., Huang X., Zhao H., Jiang X. (2020). A COVID-19 patient who underwent endonasal endoscopic pituitary adenoma resection: a case report. Neurosurgery.

[20] Jean W.C., Ironside N.T., Sack K.D., Felbaum D.R., Syed H.R. (2020). The impact of COVID-19 on neurosurgeons and the strategy for triaging non-emergent operations: a global neurosurgery study. Acta Neurochir (Wien).

[21] Khan D.Z., Bandyopadhyay S., Patel V. CSF rhinorrhoea after endonasal intervention to the anterior skull base (CRANIAL): proposal for a prospective multicentre observational cohort study. 10.1080/02688697.2020.1795622.

[22] von Elm E., Altman D.G., Egger M., Pocock S.J., Gøtzsche P.C., Vandenbroucke J.P. (2008). The strengthening the reporting of observational studies in epidemiology (STROBE) statement: guidelines for reporting observational studies. J Clin Epidemiol.

[23] Iacobucci G. (2020). COVID-19: UK lockdown is “crucial” to saving lives, say doctors and scientists. BMJ.

[24] Glasbey J.C., Bhangu A., COVIDSurg Collaborative (2021). Elective cancer surgery in COVID-19-free surgical pathways during the SARS-CoV-2 pandemic: an international, multicenter, comparative cohort study. J Clin Oncol.

[25] Nepogodiev D., Bhangu A., Glasbey J.C. (2020). Mortality and pulmonary complications in patients undergoing surgery with perioperative SARS-CoV-2 infection: an international cohort study. Lancet.

[26] Centers for Disease Control and Prevention Interim Guidelines for Clinical Specimens for COVID-19. https://www.cdc.gov/coronavirus/2019-ncov/lab/guidelines-clinical-specimens.html.

[27] Watson J., Whiting P.F., Brush J.E. (2020). Interpreting a COVID-19 test result. BMJ.

